# Of Mice and Men—The Physiology, Psychology, and Pathology of Overhydration

**DOI:** 10.3390/nu11071539

**Published:** 2019-07-07

**Authors:** Tamara Hew-Butler, Valerie Smith-Hale, Alyssa Pollard-McGrandy, Matthew VanSumeren

**Affiliations:** Division of Kinesiology, Health and Sport Studies, Wayne State University, Detroit, MI 48202, USA

**Keywords:** hydration, dehydration, hypohydration, hyponatremia, polydipsia

## Abstract

The detrimental effects of dehydration, to both mental and physical health, are well-described. The potential adverse consequences of overhydration, however, are less understood. The difficulty for most humans to routinely ingest ≥2 liters (L)—or “eight glasses”—of water per day highlights the likely presence of an inhibitory neural circuit which limits the deleterious consequences of overdrinking in mammals but can be consciously overridden in humans. This review summarizes the existing data obtained from both animal (mostly rodent) and human studies regarding the physiology, psychology, and pathology of overhydration. The physiology section will highlight the molecular strength and significance of aquaporin-2 (AQP2) water channel downregulation, in response to chronic anti-diuretic hormone suppression. Absence of the anti-diuretic hormone, arginine vasopressin (AVP), facilitates copious free water urinary excretion (polyuria) in equal volumes to polydipsia to maintain plasma tonicity within normal physiological limits. The psychology section will highlight reasons why humans and rodents may volitionally overdrink, likely in response to anxiety or social isolation whereas polydipsia triggers mesolimbic reward pathways. Lastly, the potential acute (water intoxication) and chronic (urinary bladder distension, ureter dilation and hydronephrosis) pathologies associated with overhydration will be examined largely from the perspective of human case reports and early animal trials.

## 1. Introduction

Hydration and the evolving search for an adequate universal daily water intake recommendation remains elusive [1] and somewhat contentious [2,3]. Most of the disagreement over an adequate index for fluid intake, however, likely revolves around the disparate and non-standardized metrics commonly utilized to define both normal and abnormal hydration status (HS) [4]. For example, the clinical definition of dehydration is cellular dehydration from extracellular hypertonicity [5,6] while scientists often use the term dehydration to describe the process of losing water [4]. Alternatively, the term hypohydration refers to a negative water balance [7] or state of water deficit [4]. Regardless of which hydration terminology is utilized to define HS, the vast majority of the scientific and lay literature highlights the well-recognized detrimental effects of dehydration and/or hypohydration on a variety of conditions such as kidney stones [3], obesity [8], recurrent urinary tract infections [9], cognition [10], and athletic performance [11]. 

At the opposite end of the hydration spectrum, a paucity of data exists on the topic of overhydration. In 1923, Rowntree published a series of animal and human data exploring the detrimental effects of “water intoxication” [12]. Rowntree successfully induced water intoxication (characterized by restlessness, lethargy, polyuria, diarrhea, salivation, frothing at the mouth, nausea, retching, vomiting, muscle twitching, seizures, coma and death) in dogs, cats, rabbits and guinea-pigs by rapidly administering tap or distilled water (50 mL/kg bodyweight every 30 min) rectally, intravenously, through a stomach tube and/or ureteral catheter to induce water overload [12]. Combined with similar human cases of water intoxication [13,14,15,16,17], it appears clear that extreme fluid administration in excess of excretion rates—or more modest intakes when coupled with pathological anti-diuretic hormone secretion—are indeed detrimental (and sometimes toxic) to health [12].

Thus, while the current evidence suggests that modest hypohydration and extreme overhydration have deleterious health consequences, the question remains whether modest overhydration is beneficial or detrimental to health. This review will explore the physiology, psychology, and pathology of overhydration. Both animal (mostly mice) and human studies will be detailed, with an emphasis placed upon the psychogenic polydipsia literature to more clearly evaluate: (1) long-term physiological changes associated with concomitant and sustained polyuria and (2) the putative neurogenic pathways which may differentially drive high (anxiolytic) versus low habitual fluid consumption. We will refer to the term polydipsia to represent excessive drinking (beyond regulatory need) without any known medical cause [18].

## 2. Physiology

As comprehensively described elsewhere [1,19,20,21,22], water balance is exquisitely regulated in all mammals (and some non-mammals) [22,23] in physiological defense of both osmotic balance (plasma tonicity) and circulatory volume. Plasma tonicity dictates cellular size, and is tightly regulated by a coordinated system of osmosensors, neural networks, endocrine mediators, and physiologically-driven behaviors which cooperatively serve to sustain extracellular fluid osmolality around a remarkably constant set-point of 300 mOsmol/kgH_2_O [20] (or, plasma sodium concentration ~140 mmol/L) [21]. Central to this evolutionarily stable feedback-loop controlling osmotic regulation is the kidney, whose immediate ability to retain or excrete free water is vital to the overall maintenance of fluid homeostasis [19,24]. For clinical convenience, we will refer to a plasma sodium concentration ([Na^+^]) between 135–145 mmol/L as the “normal” range for extracellular fluid osmolality/plasma tonicity since sodium is the main extracellular cation found within the plasma [21].

When modest amounts of water (or other hypotonic fluids) are ingested above osmotically-driven thirst stimulation (overhydration), osmoreceptors located within the highly vascularized circumventricular organs (CVO’s) within the brain detect a (dilutional) decrease in plasma [Na^+^] once water is absorbed into the circulation from the gastrointestinal (GI) tract [20,25]. These CVO’s, located outside of the blood brain barrier, suppress both the release of the body’s main anti-diuretic hormone, arginine vasopressin (AVP), from the posterior pituitary gland and suppress the sensation of osmotically-driven thirst to prevent further dilution of plasma [Na^+^]. Oropharyngeal receptors, activated by physical contact with ingested fluids [26,27,28], as well as gastrointestinal sensors responding to stretch receptors sensing fullness [29,30,31,32] serve to terminate drinking behavior, perhaps as an anticipatory measure to prevent the pathophysiological consequences of overdrinking (i.e., cellular swelling). In fact, recent electrophysiological and optogenic studies performed on mice confirm the presence of a distinct neuronal network, mediated by cross-talk between the brain and gastrointestinal tract, through activation of the subfornical organ within the CVO [32]. The subfornical organ appears to coordinate a variety of neuronal inputs that anticipate the homeostatic consequences of food and fluid intake well before changes in plasma tonicity are observed [32,33]. 

Functional magnetic resonance imaging (fMRI) studies further suggest that the brain senses water intake in response to thirst as “pleasant”, while overdrinking suppresses this hedonic response [34,35,36]. The alliesthesia associated with drinking while thirsty mainly activates the anterior midcingulate cortex and orbitofrontal cortex, suggesting that drinking to thirst is pleasurable and involuntary [35,36]. In contrast, continued drinking after thirst satiation (+1 L above thirst suppression) [34] activates brain areas associated with swallowing inhibition as well as cortical areas associated with unpleasantness ratings [34,35]. Activation of the motor cortex, striatum and thalamus suggests that voluntary motor activity is required to continue drinking above thirst satiation [34]. As such, drinking above thirst requires a threefold increase in volitional effort compared to drinking when thirsty [34]. Independent data collected from a clinical trial, where 316 participants with stage three chronic kidney disease were “coached” to increase daily water intake by 1–1.5 L/day, corroborate these fMRI findings and confirm that drinking above thirst is difficult and unpleasant [37]. The average increase in water intake in the participants randomized into the “coached hydration” group, could only increase their daily water intake by ~0.6 L relative to the control group [37]. Thus, the inability for free-living adults to voluntarily sustain even modest 500 mL (~2 cup) increases in daily water consumption (above thirst) underscores the strength of the central inhibitory pathways that serve to prevent the deleterious and life-threatening consequences of fluid overload.

Both thirst stimulation and AVP release are centrally coordinated in real-time by input largely from the cranial nerve system. As such, central integration of neuronal feedback from osmoreceptors (subfornical organ), baroreceptors (tenth cranial nerve), the mouth (fifth cranial nerve), tongue (seventh cranial nerve), oropharynx (ninth cranial nerve), and stomach (tenth cranial nerve) ultimately results in either the stimulation or suppression of AVP from the posterior pituitary gland [20,21,35]. AVP then regulates plasma [Na^+^]/tonicity by retaining or excreting water within the kidney collecting duct. The permeability of the kidney collecting duct increases when AVP binds to the vasopressin-2 receptors (V2R), which stimulates the insertion of aquaporin 2 (AQP2) water channels into the lumen of the kidney collecting duct [19,24]. The insertion of these AQP2 water channels allows for water molecules (otherwise destined for urinary excretion) to be reabsorbed back into the circulation when plasma tonicity is high (or circulating plasma volume low) to conserve total body water. Conversely, with overdrinking, there is central inhibition of the AVP release, which withdraws AQP2 channels from the lumen of the kidney collecting duct; thereby promoting urinary free water excretion which matches fluid ingestion beyond physiological need. 

It is important to emphasize that the neuroendocrine feedback loop coordinating fluid balance between the brain and kidney is highly conserved within the DNA (deoxyribonucleic acid) of vertebrate and invertebrate species dating back 700 million years [23]. Once released into the circulation, AVP can increase kidney collecting duct permeability within 40 s of activation of the V2R in rodent species [38]. Quantification studies of microdissected renal tubule segments, obtained from the middle part of rodent inner medullary collecting duct, estimate that there are ~12 million individual AQP2 water channels present within each kidney collecting duct cell [24]. Thus, the molecular strength and precision of the diuretic renal response to AVP suppression is powerful and allows for urinary excretion rates approximating 1 L/h, as seen in patients with diabetes insipidus [21] and compulsive water drinkers [39,40].

Interestingly enough, chronic overhydration (>3 days), triggers the downregulation of AQP2 water channels within the kidney collecting duct cells [19,24]. This phenomenon has been verified directly in a series of elegant studies performed on water-loaded rats and mice [19,24] and indirectly confirmed in human studies [41,42,43,44]. The sustained suppression of circulating AVP in response to overdrinking enhances urinary free water excretion and teleologically represents the most appropriate renal adaptation to a constant fluid intake load (polydipsia = polyuria). However, when high fluid intakes are suddenly curtailed [42,43,44], the downregulation of AQP2 water channels triggers a transient inability to reabsorb water molecules back through the kidney collecting duct in response to AVP V2R stimulation [19,24,45]. This renal insensitivity to AVP secretion augments urinary fluid losses (above intake), which is clinically characterized by an inability to concentrate urinary solutes [43,44,46,47] coupled with enhanced body water/weight loss [42]. This phenomenon of “water loading”, popularized by combat sport athletes and body builders before competition as a method to “weigh-in light” [39,45], highlights the dynamic molecular adaptability within the kidney collecting duct cells in response to chronic (>3 days) changes in water intake that have been clearly demonstrated in rodent models [19,24,48,49,50,51]. A return to regulated drinking (osmotically-driven thirst stimulation) and concomitant AVP exposure will restore AQP2 expression within collecting duct epithelium by 3–5 days [48] and reverse the physiologic nephrogenic diabetes insipidus induced by chronic water loading in both mice and men [24]. 

## 3. Psychology

At the most extreme range of overhydration, compulsive water drinking has been recognized in emotionally disturbed individuals without neurogenic (i.e., inability of secrete AVP from the posterior pituitary) or nephrogenic (i.e., kidneys resistant to AVP stimulation) diabetes insipidus [47]. Sometimes referred to as “psychogenic polydipsia” [52,53], 80% of compulsive water drinkers represent neurotic females with a history of schizophrenia [40,54], depression [14,45], and/or anxiety [53,55]. Psychogenic polydipsia in schizophrenic patients was first identified in 1936 through investigation of profuse polyuria, which eventually ceased when the polydipsia was minimized [56].

To a more modest degree of drinking, social polydipsia—or overdrinking to achieve health benefits—has become popularized within western culture. The most common “one-size-fits-all” guideline suggests that all healthy humans need to drink at least eight glasses of water daily beyond fluids obtained through foods or other beverages. This popular recommendation persists despite equivocal evidence, which supports the claim that water intake maximizes skin heath, digestion, renal, sexual, or neurological function [3,57,58]. The continued success of this advice is evidenced by robust water bottle sales, which topped 2.78 billion dollars in 2018 within the United States alone [59]. At extreme levels (>5 L daily), social polydipsia may result in profound dilation of the bladder, ureters and kidneys [60] and at worst, water intoxication (i.e., overconsumption of fluids beyond excretion rates leading to the signs and symptoms of encephalopathy) [12]. Of note, updated (2017) guidelines put forth by the European Food Safety Authority (EFSA) now defines total water intake to include all beverages consumed plus the moisture contained in foods [61].

Drinking beyond the dictates of thirst has been popularized within athletic circles to prevent the detrimental effects of hypohydration on health and performance [11]. Although guidelines have evolved to drink to minimize body weight losses (<2%) [62], other drinking guidelines recommend drinking before the onset of thirst stimulation to maintain a dilute urine (urine specific gravity <1.020) [11]. Some athletes, unfortunately, have taken this advice to extreme levels (i.e., drinking 80–100 cups of fluid during a marathon footrace [63]) and have developed water intoxication [13,64]. Additionally, water loading has become a popular practice for combat sport athletes to enhance water weight losses before weigh-ins [42] while actors participating in pornographic films have begun water loading to enhance their squirting performance abilities [58]. The prevalence of symptomatic water intoxication in prolonged endurance races remains relatively rare (<1%), however [65].

Why otherwise healthy people, outside of sports or social reasons, habitually drink high volumes of fluid [66] above physiological need remains a curious and unanswered phenomenon. Studies performed in mice exposed to chronic stress [67,68] or raised in isolation [69,70], provide psychological insight into this peculiar finding. Male C57BL/6 mice subjected to chronic social defeat stress (i.e., exposure to bigger, meaner, “bully” mice) demonstrate a distinct phenotype characterized by enhanced fluid intake [67,70] and water retention [68]. Additionally, two-month old Sabra mice [70] and adolescent male Sprague-Dawley rats [69] reared (post-weaning) in isolation developed significant polydipsia compared with control animals. Follow-up molecular and electrophysiological studies implicate the mesolimbic dopamine circuit in the manifestation of polydipsia in response to loneliness and social anxiety [67]. 

One explanation as to why anxious mice develop polydipsia, is that water intake may somehow reduce dopaminergic neuron excitability within the ventral tegmental area (VTA), or the reward area, of the brain. Of note, schizophrenia has been linked to enhanced dopaminergic receptor excitability [67,71], which likely mediates polydipsia as either a reward-seeking [67] or anxiolytic [67,68,70] behavior. Further investigations on drug-induced polydipsia are required to tease out the potential neurochemical circuits linking dopamine, reward, and drinking. Drugs that demonstrate the most promising results, include: methamphetamines (which inhibit dopamine reuptake in the brain) [72] such as 3,4 methyldioxymethamphetamine (Ecstasy) [73], agonists, which downregulate dopamine receptor 2 (DRD2) [74], first and second generation antipsychotics [75], and antidepressant medications [76]. All these drugs have been linked to hyponatremia from non-osmotic AVP secretion, but their relationship to polydipsia is under-appreciated. Of note, a variety of drugs and excipients have been shown to affect hydration status by augmenting fluid losses, affecting thirst and/or appetite, increasing intestinal permeability and/or renal reabsorption rates [7].

**Figure 1:**
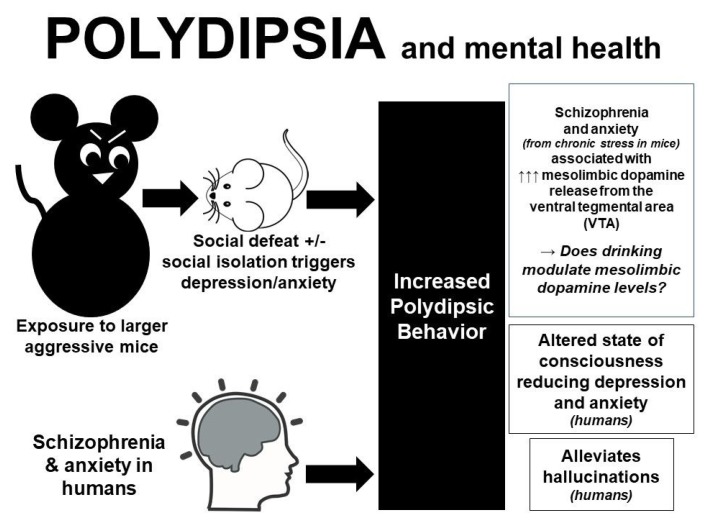
Summary of data obtained from mice and humans linking polydipsia to mesolimbic reward centers, which serve to reduce anxiety and/or signs and symptoms of psychiatric illness.

Human observations corroborate these animal findings, as most psychogenic polydipsic patients report that drinking makes them feel better [77]. Patients with hallucinations also report that drinking fluids suppress the “voices” [78], further suggesting that the act of drinking activates neural circuits associated with primitive coping mechanisms [77]. Other investigators suggest that the anterior hippocampus and nucleus accumbens are involved in polydipsia as a reward-seeking behavior in psychiatric patients [77]. Alternatively, compulsive water drinking has been documented in non-neurotic individuals seeking to achieve a drunken-like state [14,55]. For example, a 16-year-old female drank copious amounts of water because it made her feel “funny and high, like after a beer” [55] while a 46-year-old man with a history of depression ran out of money to buy beer, so drank large amounts of water because it made him feel “slightly drunk” [14]. Therefore, coupled with evidence suggesting that drinking fluids improves cognition [10], it is possible that polydipsia activates a dopaminergic reward circuit that attenuates anxiety in susceptible individuals exposed to chronic stress and/or social isolation. A summary of this proposed relationship is detailed in Figure 1. Further investigation on the potential anxiolytic effects of polydipsia, especially in females, warrants further investigation with particular regards to whether this practice is adaptive or maladaptive over the long term.

## 4. Pathology

As previously summarized, total body fluid regulation is exquisitely regulated in defense of plasma [Na^+^]/tonicity and circulating volume. Accordingly, complications from overdrinking are rare. The most common (and dire) clinical consequence from overdrinking is water intoxication, which is biochemically defined as a plasma [Na^+^] below the normal range set by the lab performing the test (usually a plasma [Na^+^] < 135 mmol/L, which is called “hyponatremia”, because hypo = low and natremia = blood sodium) [21]. As first described by Rowntree in 1923, the fatal consequences of water intoxication occur when fluid administration exceeds the capacity to excrete any fluid excess and exacerbated by AVP stimulation (water retention) [12]. Hyponatremia causes water to flow down an osmotic gradient from outside of cells to inside of cells and causes all cells within the body to swell. Hyponatremia is fatal when cerebral swelling in excess of 5–8% exceeds the rigid confines of the skull, resulting in brainstem herniation, cerebral hypoxia, and loss of vegetative functions [13,64].

Gross estimations suggest that an acute (<1 h) fluid intake around 3–4 L (~1 gallon) is enough to induce symptomatic hyponatremia in otherwise healthy individuals at rest [12,52]. Although maximal urine excretion rates allow humans to tolerate water intakes approximating 20 L per day without ill-effects [79], the actual fluid intake tolerance limit appears to be closer to 10 L per day in normal individuals [43,79]. De Wardener and Herxheimer each drank 10 L of water per day (250–500 mL every 30–60 min during waking hours) for 11 days and reported physical signs of headache, scotoma, skin coldness with pallor, and puffiness of the face [43]. These two subjects also reported that their lips felt dry (without the sensation of thirst), food was tasteless, emotional liability was high, and simple intellectual tasks became increasingly difficult during this period of enhanced water intake [43]. Of note, the pathogenic effects of overhydration are not isolated to oral intake. The first water intoxication fatality was reported in 1935, in a 50-year-old female who received 9 L of fluid over 24-h through the rectum (proctoclysis) following an otherwise uncomplicated gallbladder surgery [17]. Thus, it is difficult to commit to recommending a threshold volume of water that can be safely consumed (or administered) over time, since both the ~3 L per hour and ~10 L per day can be tolerated by some (especially athletes with high sweat rates [80]) but fatal to others. Of note, when non-osmotic AVP secretion is present, or when sodium losses are severe, modest water ingestion at rates of 1–2 L/h can induce symptomatic hyponatremia [13,21]. Non-osmotic stimuli to AVP secretion, sodium losses, a variety of drugs and excipients influencing hydration status, and type of fluids consumed are beyond the scope of this review and detailed elsewhere [7,81,82].

**Figure 2:**
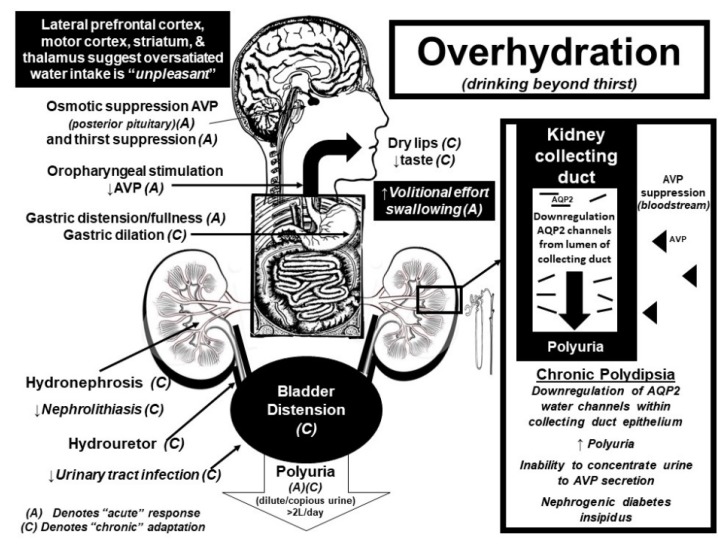
Summary diagram of acute (A) and chronic (C) physiological responses integrating potential pathologies and benefits associated with overdrinking in the satiated condition (above thirst stimulation).

**Figure 3:**
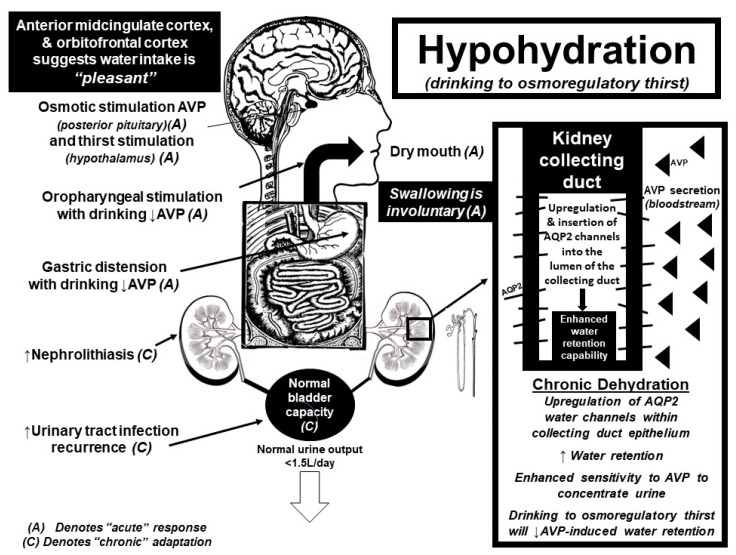
Summary diagram of acute (A) and chronic (C) physiological responses and potential pathologies associated with drinking to thirst when hypohydrated.

The body’s appropriate fluid homeostatic response to polydipsia is polyuria (i.e., excessive urine production). For individuals with normal kidney function, any excess fluid that is ingested (beyond osmoregulatory need) is promptly excreted by the body. For example, the 10 L of water ingested by De Wardener and Herxheimer resulted in a daily urine output of 10 L [43]. Polyuria is also the characteristic feature of diabetes insipidus (both neurologic and nephrogenic), whereas chronic urinary free water excretion (from AVP suppression or renal insensitivity) is counterbalanced by osmotic thirst stimulation and concomitant water intake, which matches urinary fluid losses (to maintain plasma [Na^+^] within the normal physiological range) [21,47,83]. As such, sustained polyuria has been shown to cause profound urinary tract changes such as bladder distension, dilation of the ureters, renal back pressure atrophy, hydronephrosis, traumatic rupture of the urinary tract, and renal failure [60,84,85,86,87,88]. One such case of (reversible) hydronephrosis occurred in an otherwise healthy 53-year-old female who drank 4.5–5.5 L of fluid daily over the subsequent three years to “stay healthy” and because “all her friends do so” [60]. Another possible mechanical consequence of polydipsia is gastric distension [89], which may be advantageous in those trying to lose weight (producing the sensation of stomach fullness ahead of meals) [8]. Figure 2 summarizes the acute and chronic physiological effects of overhydration while Figure 3 summarizes the acute and chronic physiological effects of water intake when hypohydrated. 

With specific regards to kidney function, individuals with a history of kidney stones (nephrolithiasis) have a reduced risk of recurrent stone formation if they consume more than 2 L of water per day [3,57]. One hypothesis is that increased water intake (>2 L/day for 12-months) reduces renal papillary density, which may precipitate calcium oxalate stone formation [90]. Conversely, excessive fluid intake may exacerbate proteinuria [91], have no effect [37] or accelerate [92] the progression of chronic renal disease. 

It has been demonstrated in a randomized-control trial that premenopausal women with a history of recurrent urinary tract infections (UTI), who drink less than 1.5 L of water daily (low volume drinkers), can reduce the recurrence rate of UTI’s from three to two episodes/year by increasing water consumption by +1.3 L/day [9]. However, increased fluid intake has not been equivocally shown to enhance skin complexion or kidney function [3,57], while data are unclear regarding constipation [57,93]. Data regarding the effect of water intake on weight loss are mixed. Some studies demonstrate positive associations between water intake, weight management and body composition [94,95,96], while others demonstrate an increase in energy intake when pre-meal water ingestion was removed [97,98]. Alternatively, a randomized control trial performed on obese and overweight adolescents did not demonstrate enhanced weight loss with increased water consumption [8]. 

It is important to note that water intake is not completely benign. Otherwise healthy individuals have died or developed significant brain swelling (hyponatremic encephalopathy) from drinking too much fluid to prevent kidney stones [99], sooth a toothache [100], dilute ingested poison [16], counter a UTI [101], treat gastroenteritis [15], and alleviate constipation [46,102], as shown in Table 1. These cases highlight the need to dampen overzealous (but well-intended) advice to “stay hydrated”. 

The potential for abnormal thirst regulation to contribute to pathological water consumption has been documented in a few select scenarios (one in humans and another in cattle). Compulsive water drinkers demonstrate abnormal thirst regulation, whereas the osmotic threshold for thirst stimulation is paradoxically lower than the osmotic threshold for AVP release [83]. Whether or not this reverse in osmotic thresholds for thirst and AVP stimulation is a cause or effect of psychogenic polydipsia remains unclear. Additionally, the animal studies suggest that most mammals will not voluntarily develop water intoxication, unless artificially induced in the laboratory to investigate hyponatremia [12,103]. The only confirmed exception are calves (and in rare instances, adult cattle) who develop fatal water intoxication only after given access to water following a period of water deprivation for reasons which remain unclear [104]. 

Finally, in contrast to the potential beneficial effects of polydipsia in healthy humans detailed elsewhere [1,4,57], fluid overload is conversely associated with an increase in mortality in unhealthy animals and humans. More specifically, hyponatremia is associated with an increased mortality rate in hospitalized dogs and cats [105]. Schizophrenic patients with polydipsia demonstrate a higher mortality rate [18] while fluid retention/overload predicts 30-day mortality rate in geriatric patients [106] while increasing morbidity and mortality in critically ill children [107]. One proposed mechanism to explain the increased mortality in compromised patients is a plausible relationship between fluid overload and inflammation, which has been observed in patients with chronic kidney disease [108,109]. Whether or not fluid overload or hyponatremia is a cause or result of disease progression remains unclear [110]. 

## 5. Conclusions

Studies performed in mice and men collectively suggest that modest overhydration results in modest urine production (which matches fluid intake volumes) in homeostatic defense of plasma tonicity and intracellular size. In the chronic condition (>3 days) sustained AVP suppression results in the downregulation of AQP2 water channels within the kidney collecting duct, which results in a transient (3–5 days) inability to concentrate urine or reabsorb kidney water back into the circulation in response to AVP stimulation. Complications from acute (>3 L/h) or chronic (5–10+ L/day) water intakes at rest are uncommon but may result in acute water intoxication or chronic urinary tract abnormalities such as urinary bladder distention, ureter dilation, and hydronephrosis. Modest overhydration (>2 L/day in sedentary individuals of average size in temperate environments) may prevent kidney stones in individuals with recurrent nephrolithiasis or reduce the number of urinary tract infections in susceptible premenopausal females. The anxiolytic effects of copious water intake on a subset of vulnerable individuals, with or without mental illness, has been demonstrated along with data suggesting that overhydration enhances cognitive function. Further studies assessing the benefits and detriments of water intake above thirst are required, as long as water intakes are not extreme and warnings of the potentially fatal consequences of water intoxication are duly noted.

## Figures and Tables

**Table 1:** Cases of hyponatremic encephalopathy (and death) in otherwise healthy people who overdrank to treat another medical condition (L = liters).

Subject	Amount of Fluid Consumed	Reason for Polydipsia	Report
Not described	3 L/20 min	Test skin elasticity	Rowntree 1923 [12]
16 yo female	20 L/day	Facial acne	Lee 1989 [55]
44 yo male	12 L/day	Kidney stones	Berry 1977 [99]
9.5 yo male	10–15 L/24 h	Soothe toothache	Pickering 1971 [100]
* 40 yo female	“plenty of water”	Dilute poison (ingested)	Sarvesvaran 1984 [16]
59 yo female	“plenty of water”	Urinary tract infection	Lee 2016 [101]
* 27 yo female	“lots of water”	Gastroenteritis	Sjoblom 1997 [15]
52 yo male	6 L/2 h + 1 L enema	Constipation	Swanson 1958 [102]
74 yo female	10–14 glasses water/day	Soften stool	Walls 1977 [46]

* fatality.
